# Factors Associated with Being on Track for Early Childhood Development in Kinshasa: A Community-Based Cross-Sectional Study

**DOI:** 10.3390/children12101329

**Published:** 2025-10-03

**Authors:** Berthold M. Bondo, Francis K. Kabasubabo, Nicaise M. Muyulu, Din-Ar B. Batuli, Gloria B. Bukasa, Paulin B. Mutombo, Pierre Z. Akilimali

**Affiliations:** 1 Mère et Enfant Barumbu General Referral Hospital, Health Zone of Kinshasa, Kinshasa P.O. Box 11850, Democratic Republic of the Congo; bmbondo900@gmail.com; 2 Patrick Kayembe Research Center, Kinshasa School of Public Health, University of Kinshasa, Kinshasa P.O. Box 11850, Democratic Republic of the Congo; francis.kabasubabo_kabengele@unilim.fr; 3 Inserm U1094, IRD UMR270, CHU Limoges, EpiMaCT-Epidemiology of Chronic Diseases in Tropical Zone, Institute of Epidemiology and Tropical Neurology, OmegaHealth, University of Limoges, 87000 Limoges, France; 4 General Referral Hospital of Mokala, Health Zone of Mokala, Kinshasa P.O. Box 11850, Democratic Republic of the Congo; nicaisemuyulu8@gmail.com; 5 General Referral Hospital of Makala, Health Zone of Selembao, Kinshasa P.O. Box 11850, Democratic Republic of the Congo; btlbopete@gmail.com; 6 Renaissance University Hospital Centre, Health Zone of Gombe, Kinshasa P.O. Box 11850, Democratic Republic of the Congo; globukasa@gmail.com; 7 Department of Nutrition, Kinshasa School of Public Health, University of Kinshasa, Kinshasa P.O. Box 11850, Democratic Republic of the Congo; paulinmutombo2004@yahoo.fr

**Keywords:** early childhood development, ECDI2030, stunting, socioeconomic status, childhood nutrition, cross-sectional survey

## Abstract

**Background/Objectives:** This study examines the associations between household socioeconomic status (SES), child nutrition, and developmental status among children aged 24–59 months in the Mont Ngafula health zone in Kinshasa. The primary research question focuses on how SES and stunting affect developmental outcomes in early childhood. **Methods:** A cross-sectional analysis was conducted involving 348 children, assessing developmental outcomes using the Early Childhood Development Index (ECDI2030). **Results:** The study found that 70.4% of children were classified as on track, with ONTRACK prevalence increasing across SES tertiles. Children who attended preschool education had higher odds of being on track. The rich tertile had higher odds of being on track than those in the poor tertile, while the middle tertile showed a weaker association. Child age categories and stunting were inversely associated with being developmentally on track. The results are consistent with multiple imputation sensitivity analyses. **Conclusions:** The study concludes that preschool attendance and a higher household socioeconomic position are strongly associated with better early developmental outcomes, while an age of 48–59 months and stunting are associated with a markedly lower likelihood of being developmentally on track. Integrated policies that reduce household poverty, promote early education, and prevent/treat early faltering growth could improve early childhood developmental trajectories.

## 1. Introduction

Early childhood represents a critical period that determines an individual’s health, education and productivity throughout their life [1]. The early years of life are characterized by rapid brain development, making children sensitive to environmental influences [2]. Evidence shows that appropriate interventions during this period can significantly improve children’s academic achievement, reduce social and economic inequalities, and promote better health outcomes in adulthood [3,4]. Worldwide, 80 million children aged 36–59 months are off track for their early childhood development (ECD) according to the former Early Childhood Development Index (ECDI), with Sub-Saharan Africa being the most affected region due to poverty and chronic malnutrition [5]. The international committee has recognized this problem as needing to be solved as a priority through Sustainable Development Goal 4 (SDG4), which calls for access to quality ECD, care, and education by 2030 [6]. UNICEF developed ECDI2030 to monitor the progress made, targeting children aged 24–59 months [7].

Despite the growing global use of ECDI2030, local evidence in Kinshasa is limited. National surveys such as the DRC MICS 2018 report an on-track prevalence of approximately 56.7% at the national level and 72.7% for Kinshasa specifically, based on the findings of the former ECDI [8]. Although this national survey provides important country benchmarks for ECD, those aggregates mask substantial within-city and within-health-zone variations, with studies showing significant variations between neighborhoods in Kinshasa [9,10,11]. Such heterogeneity limits the utility of national figures for local program planning and resource allocation.

This study addresses that gap by applying the ECDI2030 in a three-stage probability sample representative of Mont Ngafula II, an urban–rural health zone in Kinshasa. To the best of our knowledge, we provide the first sub-district ECD estimates in this health zone and directly link those local prevalence estimates with measured preschool attendance, child nutritional status, and household socioeconomic position. By presenting health zone levels, survey-weighted estimates, and multivariable associations, this study supplies actionable, locally disaggregated evidence that complements national reports such as DRC MICS 2018 and supports targeted early childhood policies at the health zone level.

This study aimed to estimate the survey-weighted prevalence of being “on track” on the ECDI among children aged 24–59 months in Mont Ngafula II and to identify factors associated with being on track.

## 2. Methodology

### 2.1. Study Design and Sampling

A cross-sectional quantitative analysis of household data was collected with a survey in Mont Ngafula health zone in Kinshasa, DRC, between 2 December and 20 December 2024. Observations with missing outcome data were excluded from the analyses, unless otherwise stated. A three-stage probability sampling strategy was used: first, five health areas were randomly selected from the Mont Ngafula II health zone sampling frame; second, within each selected health area, enumeration areas (EAs) were randomly selected proportional to size; third, within each EA, households were listed and systematically sampled. In each selected household, one eligible child (24–59 months) was randomly selected for inclusion. A sample size target of 297 was calculated, assuming an expected on-track ECD prevalence of 72.7%, a desired precision of ±5 percentage points, 95% confidence, the population of children aged 24–59 months in the health zone (11, 233), and a design effect of 1. Sampling weights were calculated as the inverse of the probability of selection at each stage (product of stage-specific selection probabilities), adjusted for non-response, and post-stratified to known population totals for child age and sex where available. In all prevalence estimates and regression analyses, we applied the survey weights and took into account the clustering at the enumeration area (EA) level, as well as the stratification by health area. For the variance estimation, we employed robust (Taylor series linearization) standard errors.

### 2.2. Measurements

Parents or caregivers completed a standardized questionnaire in French or Lingala (the local language), which was administered by trained interviewers during household visits. Seventy-five percent of respondents were mothers of the children (Table S1). The questionnaire included the UNICEF ECDI2030 items [7,12], household assets and characteristics for socioeconomic status (SES), preschool attendance (defined as any regular attendance at a formal or informal group educational program for children prior to primary school in the past 12 months), the child’s dietary diversity assessed with a 24 h dietary recall of consumption of eight food groups of the FAO [13], and household food insecurity assessed with the Household Food Insecurity Access Scale (HFIAS) [14]. Anthropometric measures (weight, length/height) were taken following the WHO field protocols and used to calculate height-for-age Z-scores (HAZ) and weight-for-height Z-scores (WHZ). Stunting was defined as HAZ < −2, and wasting was defined as WHZ < −2 [15]. Questionnaires were translated and back-translated and pilot tested prior to conducting the fieldwork. The interviewers received five days of training, and the field supervision included daily quality checks. Regarding the anthropometric measurement, weight was measured to an accuracy of 0.1 kg using a SECA electronic battery-powered personal scale. Weighing was conducted while the children were dressed in light clothing and without shoes. The scale was calibrated prior to weighing each child. Height was measured using a short measuring rod positioned horizontally, with readings taken in centimeters to an accuracy of 0.1 cm. During the measurement, the child’s head, shoulders, buttocks, knees, and heels were ensured to be in contact with the vertical board.

The primary outcome was “on track” development status measured with the ECDI2030 and coded as a binary variable ONTRACK (Yes/No). ECDI2030 assessed ECD through 20 behavioral and cognitive items that represent developmental milestones. The child achieves a milestone if the response is “yes” from items 1 to 18; any other response than “every day” for item 19, and any other response than “more often” or “much more often” for item 20 item are considered “on track” developmental status (Table 1). The minimum number of milestones that need to be achieved to be considered on track for each age group is as follows: 7 for 24 to 29 months, 9 for 30 to 34 months, 11 for 35 to 41 months, 13 for 42 to 47 months, and 15 for 48 to 59 months. A child was classified as “on track” if they achieved the minimum number of developmental milestones specified by ECDI2030 for their age group as mentioned above. We included the verification process of respondent’s understanding of the ECDI items, including any training provided to enumerators to ensure accurate reporting. Key exposures included household SES categorized into tertiles (poor, middle, rich), child age group (24–35, 36–47, 48–59 months), and child sex. Nutritional status variables were derived as binary indicators: stunted (1 = stunted, 0 = not stunted) and wasted (1 = wasted, 0 = not wasted). Additional covariates included respondent sex, caregiver education (Secondary school and above vs. lower), household size (6+), improved sanitation facilities and drinking-water sources, and a continuous individual dietary diversity score (IDDS). Dietary diversity score (DDS) collected reflects the meal from the previous day. The dietary categories include breast milk, roots and tubers, cereals, dairy products, legumes and nuts, animal proteins (meat, fish, fowl, and organ meats), eggs, fruits and vegetables rich in vitamin A, and other fruits. Each food group received a score of ‘1’ for consumption and ‘0’ for non-consumption. The comprehensive dietary variety score for each kid was calculated by aggregating the scores from all food groups, with values spanning from 0 to 8. Infants who ingested five or more food categories were deemed to satisfy the minimum dietary diversity criteria, whereas those who consumed fewer than five food groups did not fulfil the criteria. Children’s ages were collected from the respondents’ declarations or from birth certificates during the survey. To ensure inter-rater reliability among enumerators, a comprehensive training program was implemented, which included standardized protocols and detailed guidance on data collection methods. Following the training, enumerators underwent a series of assessments to evaluate their consistency and accuracy in reporting, allowing for adjustments and feedback to enhance reliability before data collection commenced. Data was collected using smartphones programmed with SurveyCTO version 2.81.3 and stored on the Kinshasa school of public health server.

### 2.3. Statistical Analysis

Sample characteristics were described with frequencies and means (unweighted) and the unweighted prevalence of ONTRACK was calculated with 95% confidence intervals. Bivariate associations between ONTRACK and categorical covariates were assessed using Pearson’s chi-square tests. Continuous predictors were compared with simple linear regression. Multivariable logistic regression was used to estimate adjusted odds ratios (aORs) and 95% confidence intervals (CIs) for factors associated with being on track (ONTRACK). The primary adjusted model included child age group, child sex, preschool attendance, IDDS, stunting and wasting indicators, parent or caregiver sex and education level, household size, SES, improved water, and sanitation. Model estimates were exponentiated to the present odds ratios. The main model and re-ran models, including SES × child sex interaction, were stored to evaluate effect modification. Model diagnostics included variance inflation factors to assess collinearity (ordinary logistic model). The mean VIF value of 3.07 indicates that, on average, the variables have low multicollinearity. Hosmer–Lemeshow goodness-of-fit, the linktest for specification, classification table measures (sensitivity, specificity, positive/negative predictive values), and the inspection of influential observations (DFBETAs) for the unweighted models. Marginal predicted probabilities and contrasts were estimated using margins and visualized with marginsplot to illustrate adjusted probabilities of ONTRACK across SES and age strata. The sensitivity analyses excluded observations with missing anthropometry measurements and multiple imputations via chained equations for anthropometric and nutrition variables with 20 imputations. Pooled estimates were obtained with the mi estimate. All statistical analyses were conducted in Stata version 17.0/SE. Statistical significance was assessed as a two-sided alpha of 0.05. The response rate was 98.0% (348/355).

### 2.4. Ethics

The survey protocol was approved by the Kinshasa School of Public Health Ethics Committee (ESP/CE/84/2025), and informed consent was obtained from the caregivers prior to participation.

## 3. Results

### 3.1. On Track for ECD Prevalence and Sample Characteristics

The sample for analysis comprised 348 children aged 24–59 months. Of these, 245 (70.4%) were classified as on track by the ECDI2030, and 103 (29.6%) were not on track. The sample was approximately balanced by sex (170 girls [48.9%]; 178 boys [51.1%]) and included children across three age groups: 24–35 months (*n* = 134, 38.5%), 36–47 months (*n* = 124, 35.6%), and 48–59 months (*n* = 90, 25.9%). The mean (SD) child age was 39.5 months (SE 0.56). The mean weight and height were 14.07 kg (SE 0.17) and 95.26 cm (SE 0.51), respectively (see Table 2).

### 3.2. Factors Associated with Being on Track for ECD

#### 3.2.1. Bivariate Associations

In unadjusted cross-tabulations, preschool attendance (Pearson chi2(1) = 11.08, *p* = 0.001) and SES (Pearson chi2(2) = 14.01, *p* < 0.001) were strongly associated with ONTRACK status. The proportion of on-track children increased across preschool attendance categories (not attended 65.34%; attended 83.51%) and SES tertiles (poor 59.5% [69/116], middle 69.8% [81/116], rich 81.9% [95/116]). Stunting (*p* = 0.013) and parent or caregiver age categories (0.046) were weakly associated with ONTRACK status. There were no significant bivariate associations between ONTRACK and child sex (*p* = 0.586) or child age group (*p* = 0.171). In a simple regression, ONTRACK was positively associated with the continuous score of SES (coefficient 0.855; 95% CI 0.398 to 1.313; *p* < 0.001).

#### 3.2.2. Multivariable Logistic Regression (Primary Model)

The primary adjusted logistic regression included 302 observations (listwise deletion for missing covariates). After adjustment, children who attended a preschool education program had higher odds of being developmentally on track compared with those who did not attend such a program (aOR 4.77, 95% CI 2.23–10.21, *p* < 0.001). Children from the rich tertile had substantially higher odds of being on track compared with children from the poor tertile (aOR 2.65, 95% CI 1.34–5.24, *p* = 0.007). Children with a middle SES had a non-statistically significant higher odds compared with those with a poor SES (aOR 1.72, 95% CI 0.93–3.19, *p* ≈ 0.080). Child age categories and stunting were inversely associated with being on track. Children aged 48–59 months had lower odds of being developmentally on track compared with those aged 24–35 months (aOR 0.26, 95% CI 0.12–0.56, *p* = 0.001). Children aged 36–47 months also had lower odds of being developmentally on track compared with those aged 24–35 months (aOR 0.55, 95% CI 0.31–0.96, *p* = 0.037). Stunted children had substantially lower odds of being developmentally on track (aOR 0.41, 95% CI 0.18–0.93, *p* = 0.034). Other covariates (child sex, child IDDS, wasting, parent or caregiver sex, parent or caregiver education, marital status, household size, drinking water source, and sanitation) were not statistically significant in the fully adjusted model (see Table 3 for full model coefficients).

#### 3.2.3. Predicted Probabilities and Interaction Analyses

**Figure 1:**
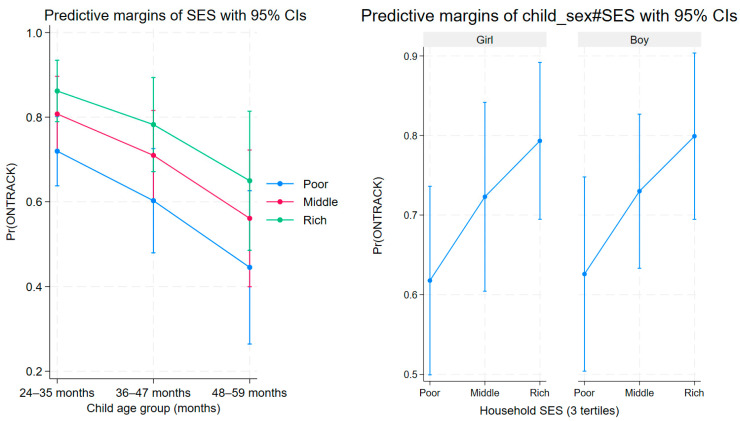
Socio-economic status and Child Development: Predictive Margins by Age and Gender. Predictive margins of child development status (Pr(ONTRACK)) vary by socioeconomic status (SES) and child age group. The probabilities of children being developmentally “on track” are depicted for Poor (blue), Middle (red), and Rich (green) categories across three age groups: 24–35 months, 36–47 months, and 48–59 months, with error bars indicating 95% confidence intervals. Additionally, interaction effects between child sex (Girl, Boy) and SES are shown, representing the ONTRACK probabilities for each sex within SES categories and including confidence intervals for reliability.

The marginal predicted probabilities of ONTRACK by SES and child age group showed a graded relationship: for 24–35-month-old children, the *p*-values for adjusted probabilities were 0.668 for poor (95% CI 0.554–0.782), 0.780 for middle (0.679–0.881), and 0.860 for rich SES status (0.784–0.935). For 36–47-month-old children, the same values were 0.565 for poor (0.440–0.691), 0.696 for middle (0.588–0.804), and 0.798 for rich SES status (0.692–0.903); and for 48–59-month-old children, these values were 0.527 for poor (0.373–0.681), 0.662 for middle (0.523–0.801), and 0.771 for rich SES status (0.652–0.891) (Figure 1). An interaction model including child_sex × SES produced no evidence of effect modification (interaction terms did not reach conventional significance) (margins plotted by sex).

#### 3.2.4. Model Performance and Diagnostics

The classification table (cut off point 0.5) indicated high sensitivity (96.3%) but low specificity (13.6%), with overall correct classification of 72.52%. The Hosmer–Lemeshow goodness-of-fit test suggested adequate calibration (Hosmer–Lemeshow χ^2^(8) = 4.25; *p* = 0.834). The linktest showed a significant _hat (*p* < 0.001) and a non-significant _hatsq (*p* = 0.918), indicating predictive information without evidence of gross specification error captured by the squared predictor. Collinearity diagnostics (uncentered VIF) indicated elevated VIF values for several variables, including “stunted” (VIF = 5.88), “marital status” (VIF = 4.63), and “meal” (VIF = 4.21), with an overall mean VIF of 3.07.

#### 3.2.5. Sensitivity Analyses

The results of the sensitivity analyses were robust. Multiple imputations (20 imputations) for anthropometry and nutrition variables produced similar directions and magnitudes of association: preschool attendance remained positively associated with ONTRACK status (mi pooled estimates: coefficient for preschool attendance = 1.63, *p* < 0.001), while child age categories and stunting remained inversely associated with ONTRACK (mi pooled estimates: coefficients for child age 36–47 months = −0.67, *p* = 0.041; child age 48–59 months = −1.30, *p* = 0.001; and stunted ≈ −0.89, *p* = 0.026). Excluding observations with missing anthropometry measurements produced qualitatively similar results.

## 4. Discussion

In this cross-sectional survey, a clear, graded relationship was found between household SES and early developmental status: children from the wealthiest households were approximately three times more likely to be developmentally on track than children from the poorest households. Concurrently, stunting was strongly independently correlated with being off track, even after adjusting for sociodemographic and household factors. Together, these results reinforce a well-established pathway linking poverty to compromised child growth and, in turn, to poorer developmental outcomes.

This study reveals several key findings that contribute significantly to the existing body of knowledge on early childhood development in the DRC. Notably, the study found that household socioeconomic status is a strong associated factor of developmental outcomes, emphasizing the need for targeted interventions. Additionally, data indicate that preschool attendance not only enhances cognitive skills but also fosters social-emotional development among children. These insights underscore the importance of contextualized research in informing public health strategies and educational policies, paving the way for future studies to explore the interplay between socioeconomic factors and developmental trajectories.

In the present study, 70.40% of children aged 24 to 59 months were developmentally on track in the Mont Ngafula II health zone. Although it is difficult to make a comparison due to the different indicators used in Sub-Saharan Africa and the DRC, this prevalence is higher than the average for Sub-Saharan Africa (55%) [5] and that in the DRC (56.7%) and similar to the prevalence in Kinshasa (72.7%) [8]. A recent assessment of ECD showed a higher prevalence than ours when using the same index in Thailand [16]. This indicates a delay in investment in ECD interventions in the DRC, but it is important to note that some areas, such as Mont Ngafula II, are making progress towards achieving SDG target 4.2 in the DRC; these areas could serve as a reference for rural and urban–rural areas to improve ECD at the national level.

Preschool attendance was strongly associated with being developmentally on track. These results are consistent with several studies [17,18,19]. This could be explained by the fact that the legal framework for the organization of preschool education in the DRC is better implemented in urban areas, where there is a proliferation of institutions and community initiatives in favor of preschool education, with a high concentration in Kinshasa and Haut Katanga [20,21].

With regard to the association between household SES and ECD, although the indicators are different and make comparison impossible, these results are consistent with those of many previous studies [19,22,23]. Several neuroscience studies have highlighted the associations between low socioeconomic status and small gray matter volume in the hippocampus, which is in turn associated with a low volume in the frontal and temporal lobes [24,25]. In contrast, a recent study in Thailand in 2024 that used the same index showed that SES was not associated with ECD [16]. This is likely due to the impact of an intervention aimed at reducing socioeconomic disparities and child poverty by providing assistance to poor families [23].

Our study indicates a negative association between a child’s age and ECD. This was similar to research performed in Afghanistan [26] and contradicts what is known in the literature, namely that the older the child, the more likely they are to be developmentally on track [7,27,28,29]. This observation in the present study could be explained by several contextual factors that have been highlighted in the literature. First, children in younger age groups receive more parental attention, particularly in terms of care and stimulation, compared with older children, who are assumed to be less vulnerable [30,31,32,33,34]. Also, younger children receive slightly more integrated health services (vaccination, CPS), providing them with access to essential health, nutrition, and development interventions [33,35,36]. The negative association between stunting and ECD, consistent with the existing literature, likely stems from nutritional deficiencies affecting brain growth, myelination, neural connectivity, and neuroplasticity, leading to delays in motor, cognitive, and socioemotional development [37,38]. In addition, children with stunted growth often exhibit apathy, reduced responsiveness, and more frequent episodes of infectious diseases, which limit their social interactions and restrict them from receiving the stimulation necessary for optimal development [4,39,40]. Finally, stunted growth often reflects conditions of poverty and food insecurity, which are accompanied by an impoverished educational environment that exacerbates developmental inequalities from early childhood [19,22]. The association observed in this study may result from common determinants, such as structural poverty or low stimulation, rather than a direct link between stunting and brain development. However, some studies suggest potential biological mechanisms that could indicate a direct effect of stunting on brain development [41,42]. It is important to approach this relationship with caution, as the evidence for causality is not universally accepted and may not be fully supported by our analysis.

For policy and programming, our findings argue for combined, equity-focused approaches. First, poverty alleviation and social protection (cash transfers, food security programs, parental leave, and childcare subsidies) can raise household capacity to provide stimulating, resource-rich environments that support development. Second, nutrition-specific interventions (early identification and treatment of undernutrition, promotion of optimal infant and young child feeding, micronutrient supplementation) and nutrition-sensitive programs (sanitation, maternal mental health support, caregiving interventions) are needed to prevent growth faltering that translates into developmental deficits. Third, integrating early stimulation and responsive caregiving and early education components into existing nutrition and primary health platforms would address both biological and psychosocial drivers of development simultaneously including older children.

Programmatic targeting should prioritize the most deprived households and children who are already stunted and older while monitoring development outcomes (not only anthropometry) to capture intervention impact. From a research perspective, longitudinal studies are needed to clarify causal sequencing and test whether combined nutrition and early learning packages produce additive or synergistic gains in developmental trajectories. Finally, implementation should include the strong monitoring and measurement of equity (disaggregated by SES, sex, and age) to ensure that interventions reach and benefit the children at the highest risk. Our findings highlight the essential need to incorporate socioeconomic determinants and preschool attendance into public health policy and early childhood development initiatives. By customizing interventions to meet the distinct needs of families across various socioeconomic levels, policymakers can improve access to quality early childhood education and support services, thereby promoting equitable developmental outcomes. This strategy fosters healthier developmental pathways in children while enhancing community resilience and well-being, so facilitating the emergence of a more robust future generation. Overall, policies, and the effective implementation of these policies that address both household poverty and early faltering growth, delivered through integrated platforms, are the most promising avenue to improve early child development at the population scale.

This research possesses certain limitations. Respondents’ declarations relied on the accuracy and honesty of the respondents in reporting the ages of the children when birth certificates were absent. Given the availability of birth certificates, respondent declaration may affect the reliability of the age data reported. Accurate age determination is crucial in studies, especially for mothers or caregivers, as self-declared ages can introduce biases and skew results. Improving age accuracy enhances research quality and applicability to policy and practice. Age affects health, education, and social services. Respondents’ educational levels and social pressures can impact their understanding of age declaration and ability to provide accurate information. Future research should improve age accuracy. Improving age accuracy is essential for better health, education, and social services. The study focuses on the nutritional status and growth of children in early childhood using specific anthropometric measures such as height, weight, and BMI. While these measures provide valuable insights, additional anthropometric details like skinfold thickness and mid-upper arm circumference could enhance our understanding of children’s health. Future studies should incorporate a broader range of anthropometric assessments to capture a more comprehensive picture of child growth and nutrition. The sample size is sufficient for first analysis, but it may not fully reflect the population’s diversity. Selection bias may arise when some groups are inadequately represented or when specific demographic segments encounter obstacles to participation. The revised inclusion requirements now encompass a wider age range, but the emphasis remains on children aged 24 months and above. The physiological and nutritional evaluations may not encompass all pertinent indicators influencing early childhood development. Variations in dietary intake, food quality, and consumption frequency may significantly influence developmental outcomes, but they are difficult to quantify thoroughly in a cross-sectional methodology. We acknowledge that the IDDS represents the previous day’s meals without adjustments for illness or holidays, which may introduce bias into the dietary diversity scores reported by enumerators. The study’s cross-sectional methodology constrains causal inferences concerning the links among different factors and developmental outcomes. Longitudinal research would yield a more comprehensive understanding of how temporal changes in age, health, and diet affect development. The reliance on respondent’s reports to verify caretaker understanding of the ECDI items presents a limitation, as it may lead to discrepancies in responses due to subjective interpretations and potential biases. This could affect the accuracy of the data collected and the overall reliability of the findings. While our findings indicate that adequate DDS did not correlate with developmental status, it is important to acknowledge that existing literature suggests a significant relationship between DDS and child development, particularly in relation to the consumption of animal source foods. This discrepancy may warrant further investigation to understand the contextual factors influencing these outcomes and to explore the potential benefits of animal source foods, which could be analyzed separately from overall DDS.

Collinearity diagnostics (uncentered VIF) indicated elevated VIF values for some variables, including “stunted” (VIF = 5.88), “marital status” (VIF = 4.63), and “meal” (VIF = 4.21), with an overall mean VIF of 3.07. These findings suggest potential multicollinearity issues for these variables, which may affect the stability and interpretation of the model’s coefficients.

## 5. Conclusions

This study highlights the critical role of preschool attendance and higher household SES in fostering positive early childhood development outcomes within our representative sample. Specifically, we found that children from higher SES backgrounds and those attending preschool exhibited significantly improved developmental metrics, while factors such as child age and stunting were linked to poorer outcomes. These results reinforce the established connections between poverty, growth faltering, and developmental delays, emphasizing the urgent need for integrated interventions that address both nutrition and early childhood education, particularly for the most disadvantaged households.

Furthermore, our findings reveal pronounced disparities in early development outcomes within Kinshasa, necessitating tailored strategies that consider the unique socioeconomic landscapes of different neighborhoods. We recommend that future research should explore the effectiveness of context-specific interventions and their impact on child development. Additionally, further studies should aim to investigate the long-term effects of preschool attendance and socioeconomic interventions on developmental trajectories, thereby informing public health policies and educational frameworks designed to support vulnerable populations.

## Figures and Tables

**Table 1:** Development domains, subdomains, and stages of the Early Childhood Development Index 2030 (ECDI2030) [7].

Domain	Subdomain	Milestones
Health	Gross motor skills	Walks on an uneven surface
		2. Jumping with both feet
	Personal care	3. Dresses him/herself
	Fine motor skills	4. Fastens/unfastens buttons
Learning	Expressive language	5. Says 10 or more words
		6. Says sentences of 3 or more words
		7. Says sentences of 5 or more words
		8. Uses “I”, “you”, “she”, or “he” correctly
	Reading and writing	9. Names objects consistently
		10. Recognizes 5 letters of the alphabet
	Pre-writing	11. Writes his/her name
	Numeracy	12. Knows numbers 1 to 5
		13. Gives correct amount (3 objects)
		14. Counts to 10
	Executive function	15. Does an activity without asking for help or giving up
Psychosocial well-being	Emotional skills	16. Asks about familiar people
		17. Offers to help
	Social skills	18. Gets along well with other children
	Internalized behavior	19. Seems to be sad or depressed
	Externalized behavior	20. Kicks, bites, or hits

**Table 2:** Sociodemographic characteristics overall and according to early childhood development.

Characteristics	Total (Column) *n* (%) or Mean (SD)	Early Childhood Development (Row)		
		On track *n* (%) or Mean (SD)	Not on Track *n* (%) or Mean (SD)	*p*-Value
	348 (100.00)	245 (70.40)	103 (29.60)	
Children’s characteristics				
Sex				0.613
Boy	178 (51.15)	123 (69.10)	55 (30.90)	
Girl	170 (48.85)	122 (71.76)	48 (28.24)	
Age (in months)				0.156
24–35	134 (38.51)	102 (76.12)	32 (23.88)	
36–46	124 (35.63)	84 (67.74)	40 (32.26)	
47–59	90 (25.86)	59 (65.56)	31 (34.44)	
Age (continuous)	39.53 (10.04)	39.27 (10.01)	40.16 (10.12)	0.453
Preschool education *				0.001
No	251 (72.13)	164 (65.34)	87 (34.66)	
Yes	97 (27.87)	81 (83.51)	16 (16.49)	
Food diversity				0.401
Inadequate	127 (36.49)	85 (66.93)	42 (33.07)	
Adequate	221 (63.51)	160 (72.40)	61 (27.60)	
Weight (Kg)	14.08 (3.01)	14.19 (2.88)	13.83 (3.27)	0.336
Height (Cm) *	95.38 (9.10)	96.15 (8.16)	93.64 (10.77)	0.024
Stunting (*n* = 313) *				0.028
No	275 (87.86)	199 (72.36)	76 (27.64)	
Yes	38 (12.14)	20 (52.63)	18 (47.37)	
Wasting (*n* = 304)				0.874
No	272 (89.47)	191 (70.22)	81 (29.78)	
Yes	32 (10.53)	23 (71.88)	9 (28.12)	
Underweight (*n* = 314)				0.250
No	278 (85.54)	196 (70.50)	82 (29.50)	
Yes	36 (11.46)	22 (61.11)	14 (38.89)	
Parents or caregivers’ characteristics				
Sex				0.720
Female	315 (90.52)	221 (70.16)	94 (29.84)	
Male	33 (9.48)	24 (72.73)	9 (27.27)	
Age (in years)				0.088
<30	131 (37.64)	84 (64.12)	47 (35.88)	
≥30	217 (62.36)	161 (74.19)	56 (25.81)	
Education level				0.995
Low	142 (40.80)	100 (70.42)	42 (29.58)	
Secondary school or above	206 (59.20)	149 (70.39)	61 (29.61)	
Occupation				0.990
Unemployed	59 (16.09)	39 (69.64)	17 (30.36)	
Housewife	78 (22.41)	55 (70.51)	23 (29.49)	
Employee	214 (61.49)	151 (70.56)	63 (29.44)	
Marital status				0.195
Alone	86 (24.71)	55 (63.95)	31 (36.05)	
Married/in union	262 (75.29)	190 (72.52)	72 (27.48)	
Household characteristics				
Household size				0.572
<6	141 (40.52)	97 (68.79)	44 (31.21)	
≥6	207 (59.48)	148 (71.50)	59 (28.50)	
Socioeconomic status *				0.002
Poor	116 (33.33)	69 (59.48)	47 (40.52)	
Middle	116 (33.33)	80 (68.97)	36 (31.03)	
Rich	116 (33.33)	96 (82.76)	20 (17.24)	
Food security level				0.402
Food insecurity	287 (82.47)	199 (69.34)	88 (30.66)	
Food security	61 (17.53)	46 (75.41)	15 (24.59)	
Source of drinking water				0.500
Unimproved	6 (1.72)	5 (83.33)	1 (16.67)	
Improved	342 (98.28)	240 (70.18)	102 (29.82)	
Sanitation				0.285
Unimproved	110 (31.64)	72 (65.45)	38 (34.55)	
Improved	238 (68.36)	173 (72.69)	65 (27.31)	
Hand-washing point				0.514
Absent or present without soap and water	124 (35.63)	85 (68.55)	39 (31.45)	
Present with water	224 (64.37)	160 (71.43)	64 (28.57)	
Number of meals per day				0.233
<3	277 (79.60)	190 (68.59)	87 (31.41)	
≥3	71 (20.40)	55 (77.46)	16 (22.54)	

*: variables statistically significant.

**Table 3:** Full adjusted model examining child, parent or caregiver, household characteristics, and early childhood development, *n* = 302.

Variable	aOR	CI 95%	*p*-Value
Child sex (girl = ref)			
Boy	1.04	0.55–1.98	0.900
Child age (24–35 months = ref) *			
36–47 months	0.55	0.31–0.96	0.037
48–59 months	0.26	0.12–0.56	0.001
Preschool education attendance (no = ref) *			
Yes	4.77	2.23–10.21	<0.001
IDDS (inadequate food diversity = ref)			
Adequate food diversity	1.23	0.63–2.40	0.528
Stunting (no = ref) *			
Yes	0.41	0.18–0.93	0.034
Wasting (no = ref)			
Yes	1.19	0.43–3.35	0.724
Parent or caregiver age (under 30 years = ref)			
30 years and above	1.60	0.74–3.46	0.220
Parent or caregiver education level (low = ref)			
Secondary school and above	0.85	0.43–1.71	0.647
Marital status (alone = ref)			
Married or in union	1.40	0.67–2.93	0.356
Household size (under 6 = ref)			
6 and more	1.20	0.68–2.13	0.510
SES (poor = ref) *			
Middle	1.72	0.93–3.19	0.080
Rich	2.65	1.34–5.24	0.007
Drinking water source (unimproved = ref)			
Improved	0.78	0.07–8.63	0.830
Sanitation (unimproved = ref)			
Improved	1.34	0.69–2.63	0.376

*: variables statistically significant.

## Data Availability

The data used for this publication and the do.file can be made available upon reasonable request to the corresponding author.

## Supplementary Materials

The following supporting information can be downloaded at: https://www.mdpi.com/article/10.3390/children12101329/s1, Table S1. Relationship between respondent and children.
