# Supplementary material for: Factors Associated with Being on Track for Early Childhood Development in Kinshasa: A Community-Based Cross-Sectional Study
Source: Children (Basel). 2025 Oct 3;12(10):1329. doi: 10.3390/children12101329 (PMC12563683; doi:10.3390/children12101329)
Supplement: Supplementary file 1 [file children-12-01329-s001.zip › children-3883641-supplementary.pdf]

Table S1. Relationship between respondents and children

	n	%
Mother	260	74,7
Father	22	6,3
Grand Mother/Grand Father	30	8,6
Sister/Brother	6	1,7
Aunt/Uncle	28	8,0
Cousin/Domestic servant	2	0,6
